# Chloral in Asthmatic Bronchitis

**Published:** 1871-02

**Authors:** 


### Chloral in Asthmatic Bronchitis.

Dr. Caspar Morris says he was recently in attendance upon a lady who suffers from frequently recurring attacks of bronchitis, with asthma. The skin was hot, the frequency and difficulty of respiration very great, the râles loud and musical, and the secretion very profuse, so that the mucus could be poured from the cup in an abundant, ropy stream. His attention had been arrested by the account recently published, that the hydrate of chloral might be of service. He ordered five grains in one fluidrachm of the syrup of lactucarium of Aubergier, to be repeated in two hours, if required. The two doses afforded entire relief; and she has found great comfort since from a single dose taken at bed-time; a good night's rest being secured by it. He mentions it as a valuable aid in the treatment of this intractable and distressing disease.—*American Journal of Medical Science.*

